# Noninvasive amide proton transfer magnetic resonance imaging in evaluating the grading and cellularity of gliomas

**DOI:** 10.18632/oncotarget.13970

**Published:** 2016-12-15

**Authors:** Yan Bai, Yusong Lin, Wei Zhang, Lingfei Kong, Lifu Wang, Panli Zuo, Ignacio Vallines, Benjamin Schmitt, Jie Tian, Xiaolei Song, Jinyuan Zhou, Meiyun Wang

**Affiliations:** ^1^ Department of Radiology, Zhengzhou University Peoples Hospital & Henan Provincial Peoples Hospital, Zhengzhou, Henan, China; ^2^ Cooperative Innovation Center of Internet Healthcare & School of Software and Applied Technology, Zhengzhou University, Zhengzhou, Henan, China; ^3^ Department of Radiology, Ren Ji Hospital of Shanghai Jiao Tong University, Shanghai, China; ^4^ Department of Pathology, Zhengzhou University Peoples Hospital & Henan Provincial Peoples Hospital, Zhengzhou, Henan, China; ^5^ MR Collaborations NE Asia, Siemens Healthcare China, Beijing, China; ^6^ MR Collaborations NE Asia, Siemens Healthcare China, Shanghai, China; ^7^ Imaging & Therapy Systems Division, Siemens Healthcare Australia, Brisbane, Australia; ^8^ Institute of Automation, Chinese Academy of Sciences, Beijing, China; ^9^ Department of Radiology, Johns Hopkins University, Baltimore, MD, USA

**Keywords:** amide proton transfer, glioma, grading, Ki-67, magnetic resonance imaging

## Abstract

Using noninvasive magnetic resonance imaging techniques to accurately evaluate the grading and cellularity of gliomas is beneficial for improving the patient outcomes. Amide proton transfer imaging is a noninvasive molecular magnetic resonance imaging technique based on chemical exchange saturation transfer mechanism that detects endogenous mobile proteins and peptides in biological tissues. Between August 2012 and November 2015, a total number of 44 patients with pathologically proven gliomas were included in this study. We compared the capability of amide proton transfer magnetic resonance imaging with that of noninvasive diffusion-weighted imaging and noninvasive 3-dimensional pseudo-continuous arterial spin imaging in evaluating the grading and cellularity of gliomas. Our results reveal that amide proton transfer magnetic resonance imaging is a superior imaging technique to diffusion-weighted imaging and 3-dimensional pseudo-continuous arterial spin imaging in the grading of gliomas. In addition, our results showed that the Ki-67 index correlated better with the amide proton transfer-weighted signal intensity than with the apparent diffusion coefficient value or the cerebral blood flow value in the gliomas. Amide proton transfer magnetic resonance imaging is a promising method for predicting the grading and cellularity of gliomas.

## INTRODUCTION

Glioma is the most common primary tumor in the brain and is classified into four grades based on the World Health Organization (WHO) guidelines [1]. Accurate grading of a glioma is fundamental in order to determine the treatment strategy. Ki-67 index is a biomarker of cellularity in gliomas, which is elevated by increasing cellular density [2]. Using noninvasive magnetic resonance imaging (MRI) techniques to accurately evaluate the grading and cellularity of gliomas is beneficial for improving the patient outcomes.

Amide proton transfer (APT) imaging is a noninvasive molecular MRI technique based on chemical exchange saturation transfer mechanism that detects endogenous mobile proteins and peptides in biological tissues [3–5]. Preliminary studies have shown that APT-weighted (APTw) signal intensity could serve as a new imaging biomarker, by revealing significantly higher signal intensities in the high-grade gliomas compared with the low-grade gliomas [6–9].

Diffusion-weighted imaging (DWI) is a noninvasive MRI technique to describe the diffusion of water molecules. The apparent diffusion coefficient (ADC) derived from DWI has been widely used to grade gliomas and reflects tumor cellularity [10, 11]. However, ADC is not reliable enough for assessing the grading and cellular density of gliomas [12–14].

3-dimensional (3D) pseudo-continuous arterial spin labeling (pCASL) imaging is a noninvasive perfusion MRI method that allows quantification of cerebral blood flow (CBF) by using magnetic tagging of the arterial blood-water protons as an endogenous tracer. Currently, the pCASL tagging method provides the best signal-to-noise ratio [15]. The previous study has been used the 3D pCASL MRI method to evaluate the grading of gliomas [16].

To the best of our knowledge, no previous study has investigated whether APT MRI could improve the predictions of grading and cellularity in gliomas compared with DWI and 3D pCASL imaging. The purpose of this study was to compare the capability of APT MRI with that of DWI and 3D pCASL imaging in evaluating the grading and cellularity of gliomas.

## RESULTS

**Figure 1:**
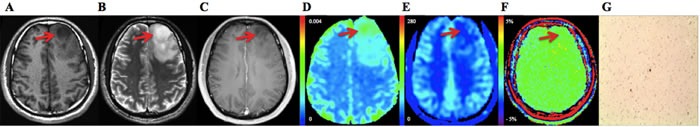
A 25-year-old male patient with astrocytoma (WHO grade 2) in the left frontal lobe The tumor has peritumoral edema. The solid tumor components (arrows) show hypointense signals on T1w image **A.** and hyperintense signals on T2w image **B.** No enhancement is revealed on post-gadolinium T1w image **C.** ADC map **D.** shows increased value. Both CBF map **E.** and APT map **F.** demonstrate no increasements of the values. Ki-67 immunohistostaining map **G.** shows no obvious increasement of the expression (original magnification, ×100).

**Figure 2:**
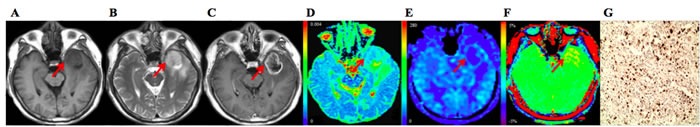
A 52-year-old female patient with astrocytoma (WHO grade 3) in the left temporal lobe The tumor (arrows) shows hypointense signals on T1w image **A.** and heterogeneous hyperintense signals on T2w image **B.** Post-gadolinium T1w image demonstrates an irregular ring-enhanced tumor with central no enhancement **C.** ADC map **D.** shows heterogeneous values. CBF map **E.** shows ring increased values and no increasement of the value in the central tumor. APT map **F.** demonstrates heterogeneous increased values. Ki-67 immunohistostaining map **G.** shows increasement of the expression (original magnification, ×100).

**Figure 3:**
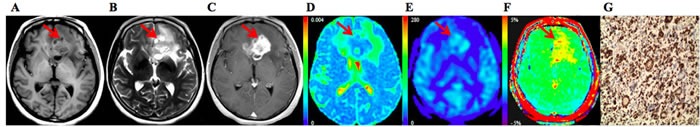
A 59-year-old male patient with glioblastoma (WHO 4 grade) in the left frontal lobe The tumor (arrows) shows heterogeneous hypointense signals on T1w image **A.** and heterogeneous hyperintense signals on T2w image **B.**Heterogeneous enhancement is noted on post-gadolinium T1w image **C.** ADC map **D.** shows decreased values. Both CBF map **E.** and APT map **F.** demonstrate increased values. Ki-67 immunohistostaining map **G.** shows obvious increasement of the expression (original magnification, ×100).

**Figure 4:**
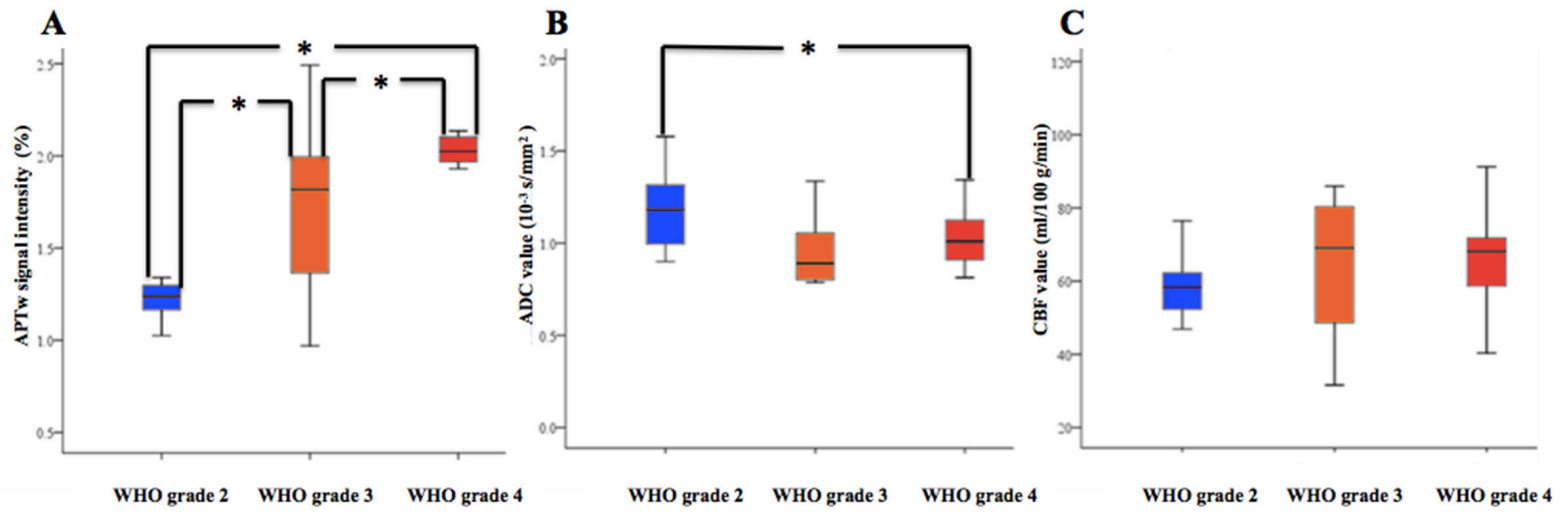
Box and whisker plots of the APTw signal intensity A., ADC value B. and CBF value C. in the WHO grade 2, 3 and 4 gliomas. * Values are significant with Bonferroni corrections for multiple comparisons (P < 0.016).

Table 1 quantitatively lists the APTw signal intensity, ADC value, CBF value and Ki-67 index among the WHO grade 2, 3 and 4 gliomas. Table 2 lists the information of included patients. Figures 1, 2 and 3 showed the manifestations of WHO grade 2, 3 and 4 gliomas on T1-weighted (T1w) images, T2-weighted (T2w) images, post-gadolinium T1w images, ADC maps, CBF maps, APT maps and Ki-67 immunohistostaining maps, respectively. Figure 4 showed the box and whisker plots of the APTw signal intensity (Figure 4A), ADC value (Figure 4B) and CBF value (Figure 4C) in the WHO grade 2, 3 and 4 gliomas.

**Table 1:** The APTw signal intensity, ADC value, CBF value and Ki-67 index (mean ± standard deviation) in the WHO grade 2, 3 and 4 gliomas

	APTw signal (%)	ADC (10^-3^ s/mm^2^)	CBF (ml/100 g/min)	Ki-67
WHO grade 2 gliomas (n = 18)	1.25 ± 0.17	1.24 ± 0.24	58.3 ± 8.2	0.19 ± 0.11
WHO grade 3 gliomas (n = 10) WHO grade 4 gliomas (n = 16) *P* values between WHO grade 2 and 3 glimas *P* values between WHO grade 2 and 4 glimas *P* values between WHO grade 3 and 4 glimas	1.71 ± 0.45 2.05 ± 0.18 0.005* < 0.001* 0.015*	1.04 ± 0.24 1.05 ± 0.16 0.024 0.014* 0.461	63.9 ± 19.6 69.2 ± 18.6 0.874 0.023 0.213	0.36 ± 0.12 0.61 ± 0.12 0.001* < 0.001* < 0.001*

Abbreviations: APTw, amide proton transfer-weighted; ADC, apparent diffusion coefficient; CBF, cerebral blood flow; WHO, World Health Organization. * Values are significant with Bonferroni corrections for multiple comparisons (P < 0.016).

**Table 2:** The information of included patients

Document number	Gender	Age (years)	Grade (WHO criteria)	Histology	Tumor size Maximal cross-sectional diameters (mm × mm)	Tumor location
1	Female	38	2	astrocytoma	53 × 49	Right frontal lobe
2	Female	57	4	glioblastoma	67 × 62	Right temporal lobe
3	Male	59	4	glioblastoma	54 × 41	Left frontal lobe
4	Female	52	3	oligoastrocytoma	42 × 50	Right temporal lobe
5	Female	62	4	glioblastoma	45 × 52	Left frontal lobe
6	Male	48	4	glioblastoma	36 × 36	Right parietal lobe
7	Male	43	2	astrocytoma	41 × 44	Left temporal lobe
8	Female	46	3	oligoastrocytoma	42 × 41	Left parietal lobe
9	Male	63	3	oligodendroglioma	53 × 64	Right frontal lobe
10	Male	54	4	glioblastoma	46 × 43	Right frontal lobe
11	Male	68	2	oligodendroglioma	52 × 49	Right parietal lobe
12	Male	57	4	glioblastoma	42 × 39	Left temporal lobe
13	Female	58	4	glioblastoma	47 × 43	Left temporal lobe
14	Male	54	4	glioblastoma	34 × 37	Right parietal lobe
15	Male	25	2	astrocytoma	34 × 31	Left frontal lobe
16	Male	66	4	glioblastoma	43 × 40	Left occipital lobe
17	Female	47	3	oligoastrocytoma	42 × 56	Right occipital lobe
18	Female	52	3	astrocytoma	33 × 36	Left temporal lobe
19	Female	50	2	oligoastrocytoma	45 × 51	Right parietal lobe
20	Male	49	4	glioblastoma	39 × 53	Right temporal lobe
21	Male	49	4	glioblastoma	61 × 54	Right parietal lobe
22	Female	41	2	oligoastrocytoma	38 × 43	Left frontal lobe
23	Female	40	2	astrocytoma	22 × 27	Left temporal lobe
24	Male	35	2	astrocytoma	47 × 42	Right temporal lobe
25	Male	60	4	glioblastoma	47 × 51	Left occipital lobe
26	Male	34	2	astrocytoma	26 × 31	Right frontal lobe
27	Male	43	3	oligodendroglioma	58 × 72	Left frontal lobe
28	Female	27	2	oligoastrocytoma	48 × 52	Right parietal lobe
29	Male	63	4	glioblastoma	68 × 63	Right temporal lobe
30	Male	37	3	astrocytoma	64 × 59	Left temporal lobe
31	Female	54	2	astrocytoma	38 × 41	Left temporal lobe
32	Female	50	2	astrocytoma	36 × 34	Right frontal lobe
33	Male	41	4	glioblastoma	39 × 53	Left parietal lobe
34	Female	29	2	astrocytoma	34 × 34	Right parietal lobe
35	Female	45	2	astrocytoma	49 × 47	Right temporal lobe
36	Female	68	3	astrocytoma	42 × 49	Left temporal lobe
37	Male	38	2	astrocytoma	31 × 34	Left frontal lobe
38	Female	69	4	glioblastoma	46 × 48	Left frontal lobe
39	Female	51	2	oligodendroglioma	41 × 36	Left parietal lobe
40	Female	56	2	astrocytoma	52 × 57	Right temporal lobe
41	Male	65	3	astrocytoma	51 × 44	Left parietal lobe
42	Female	51	2	astrocytoma	22 × 32	Left frontal lobe
43	Female	45	4	glioblastoma	42 × 40	Left temporal lobe
44	Female	44	3	astrocytoma	50 × 47	Right frontal lobe

After Bonferroni corrections for multiple comparisons, the APTw signal intensities were significantly different between the WHO grade 2 and 3, 2 and 4, and 3 and 4 gliomas (U = 32, 1 and 24, respectively; P = 0.005, P < 0.001 and P = 0.015, respectively). The ADC values were significant higher in the WHO grade 2 gliomas than those in the WHO grade 4 gliomas (U = 73, P = 0.014), whereas there were no significant differences between the WHO grade 2 and 3, and 3 and 4 gliomas (U = 43 and 66, respectively; P = 0.024 and 0.461, respectively). The CBF values were not significantly different between the WHO grade 2 and 3, 2 and 4, and 3 and 4 gliomas (U = 77, 78 and 64, respectively; P = 0.874, 0.023 and 0.213, respectively). The Ki-67 indexes were significantly different between the WHO grade 2 and 3, 2 and 4, and 3 and 4 gliomas (U = 27, 3 and 11, respectively; P = 0.001, P < 0.001 and P < 0.001, respectively).

**Figure 5:**
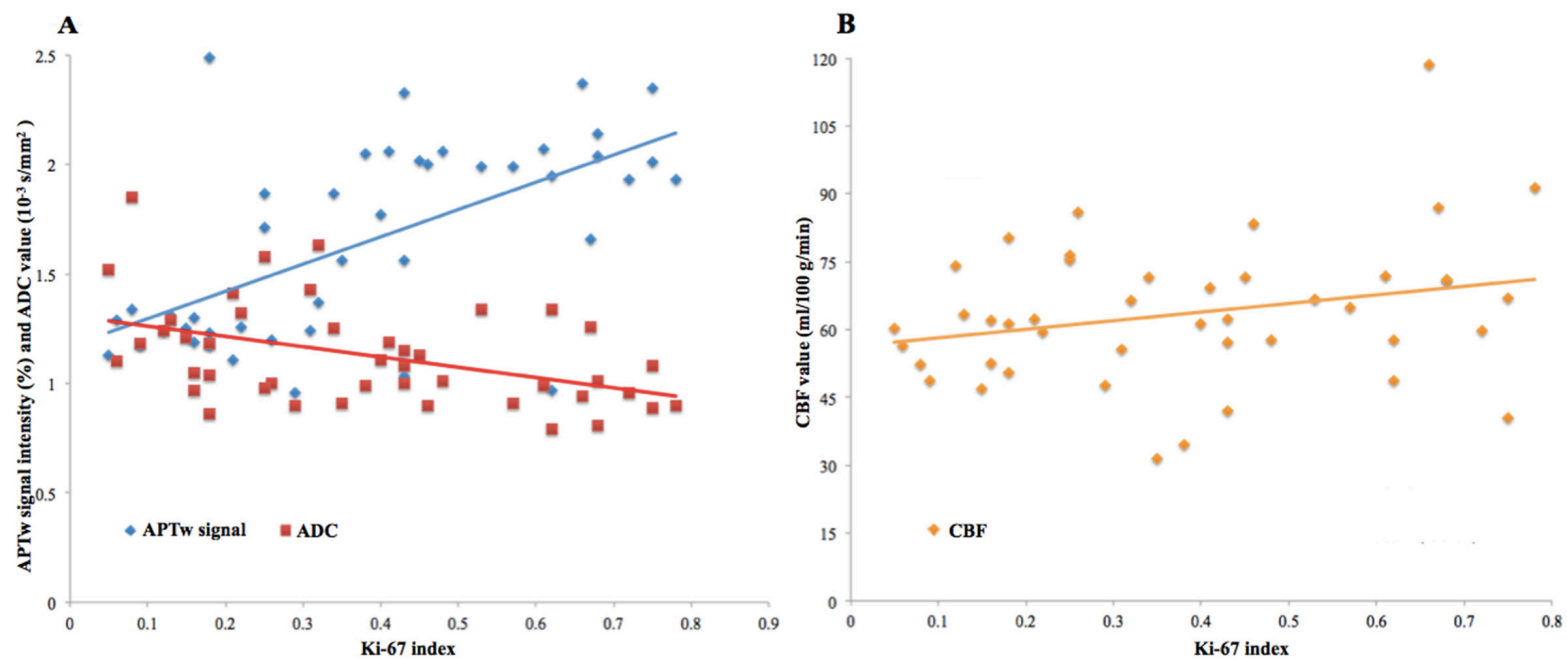
**A.** The correlation coefficient between the APTw signal intensity and the Ki-67 index (r = 0.597, P < 0.001) is significantly higher than that between the ADC value and the Ki-67 index (r = -0.441, P = 0.003) in the gliomas (P < 0.01). B. The CBF value is not significantly correlated with the Ki-67 index (r = 0.245, P = 0.109) in the gliomas.

There was a significant correlation coefficient between the APTw signal intensity and the Ki-67 index (r = 0.597 [0.357 to 0.781 with 95% confidence interval], P < 0.001), and between the ADC value and the Ki-67 index (r = -0.441 [-0.669 to -0.179 with 95% confidence interval], P = 0.003) in the gliomas. Moreover, the correlation coefficient between the APTw signal intensity and the Ki-67 index was significantly higher than that between the ADC value and the Ki-67 index in the gliomas (P < 0.01) (Figure 5A). The CBF value was not significantly correlated with the Ki-67 index (r = 0.245 [-0.045 to 0.516 with 95% confidence interval], P = 0.109) in the gliomas (Figure 5B).

**Figure 6:**
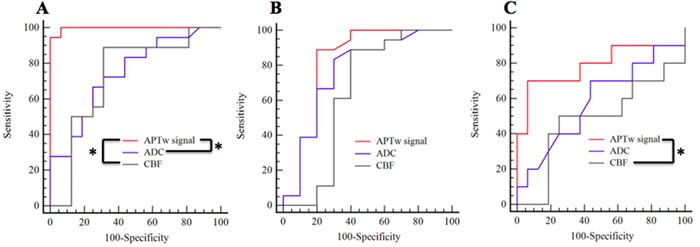
ROC curves for the APTw signal, ADC and CBF in distinguishing WHO grade 2 gliomas from WHO grade 4 gliomas A., WHO grade 2 gliomas from WHO grade 3 gliomas B. and WHO grade 3 gliomas from WHO grade 4 gliomas C. * Values are significant (P < 0.05).

The areas under the receiver operating characteristic (ROC) curves (AUCs) were analyzed as measures for the grading of gliomas. The AUC of the APTw signal intensity (0.997; 0.890 to 1.000 with 95% confidence interval) was significantly greater than that of the ADC value (0.745; 0.567 to 0.878 with 95% confidence interval) (P = 0.002) and the CBF value (0.729; 0.550 to 0.866 with 95% confidence interval) (P = 0.002) in the differentiation of the WHO grade 2 and 4 glimas (Figure 6A). There were no significantly differences between the AUCs of the APTw signal intensity (0.825; 0.635 to 0.941 with 95% confidence interval) and the ADC value (0.767; 0.569 to 0.904 with 95% confidence interval) (P > 0.05), and between the AUCs of the APTw signal intensity and the CBF value (0.644; 0.442 to 0.815 with 95% confidence interval) (P > 0.05) for the grading of WHO grade 2 and 3 gliomas (Figure 6B). For discriminating the WHO grade 3 gliomas from the WHO grade 4 gliomas, the AUC of the APTw signal intensity (0.788; 0.584 to 0.921 with 95% confidence interval) was significantly greater than that of the CBF value (0.481; 0.283 to 0.684 with 95% confidence interval) (P = 0.029), while there were no significantly differences between the AUCs of the APTw signal intensity and the ADC value (0.584; 0.376 to 0.772 with 95% confidence interval) (P > 0.05) (Figure 6C).

The intraclass correlation coefficients between two independent radiologists (Y.B. and M.W., with 9 and 18 years of experience, respectively) for the calculations of the APTw signal intensities, ADC values and CBF values were 0.919, 0.845 and 0.909, respectively. The intraclass correlation coefficient between two independent pathologists (L.W. and L.K., with 14 and 23 years of experiences, respectively) for the calculations of the Ki-67 indexes was 0.874.

## DISCUSSION

In our present study, the results showed that the APTw signal intensity was significantly different between the WHO grades 2, 3 and 4 gliomas. However, the ADC value and CBF value had inconsistently significant differences between the WHO grades 2, 3 and 4 gliomas. In addition, our results demonstrated that the Ki-67 index correlated better with the APTw signal intensity than with the ADC value or the CBF value in the gliomas.

Our results support that APT MRI is a valuable tool in evaluating the grading and cellularity of gliomas. Previous studies reported that the high-grade gliomas had higher concentration of mobile proteins and peptides than the low-grade gliomas [6–9]. In addition, APT MRI can provide contrast based on the cellular mobile proteins and peptides [3, 4].Thus, APTw signal intensity has the capability to represent the cellular density of gliomas [7]. Park et al. [8] consider that APT MRI is a promising in vivo imaging method for quantifying the cellular proliferation of gliomas. The higher APTw signal intensity in the higher-grade gliomas may be attributed to the denser cellularity in the solid components of these tumors in comparison with the lower-grade gliomas. It is worth noting that the APTw contrast visualized at around 3.5 ppm may include other contributions beside mobile proteins and peptides such as the effect of pH on proton exchange and nuclear Overhauser effect (NOE) [4, 17]. The pH environment changes the amide proton exchange rate, which influences the APTw contrast [3]. However, the effect of pH on the APTw signal intensities of gliomas should be negligible because only minimally increased intracellular pH was detected in gliomas in comparison to the normal brain tissues [18]. NOE signal entangled APTw contrast in MTR asymmetry analysis by lower signal in tumor at the unfiled of the water resonance (-2.5 to -5 ppm). However, the NOE signal can be clearly detected at lower saturation powers, while the APT signal is maximized at relatively higher saturation powers. In this study, the average saturation power of 2.0 µT was used to minimize the NOE effects for APT MRI [17]. In addition, the NOE effect is usually ignorable at 3-T MRI, but the effect is very high at 7-T MRI [17]. Furthermore, the cystic changes within the gliomas contain many mobile proteins and show high APTw signal intensities, which may lead to errors [6]. Therefore, we delineated the ROIs without the areas of necrosis and cystic degeneration on the T2w images.

In addition, our results showed that the ADC value was significantly different between the WHO grade 2 and 4 gliomas. This finding is consistent with some of the previous studies demonstrating that the ADC value representing cellularity of gliomas is significantly lower in the high-grade gliomas than that in the low-grade gliomas [10, 11]. However, our current results also showed that there were no differences in the ADC values between the WHO grade 2 and 3, and grade 3 and 4 gliomas. Indeed, some previous reports have demonstrated no significant difference in the ADC values between the high-grade and low-grade gliomas [12, 19]. Moreover, Sadeghi et al. [13] reported that the ADC value was not correlated with the cellular density of gliomas. One possible explanation for those contradictory results is that the higher tumor cellularity and vascularity in the higher-grade gliomas [11, 19] may affect the DWI signal attenuation in an opposite way [20–22]. Therefore, the ADC value has limitations in evaluating the grading and cellularity of gliomas.

The results of our present study demonstrated that there were no differences in the CBF values obtained from 3D pCASL MRI between the WHO grade 2, 3, and 4 gliomas. Our finding was consistent with the previous study reported by Roy et al. [16], which showed that 3D pCASL MRI could not be regarded as a reliable technique in the grading of gliomas. The CBF value quantified by pCASL method was influenced by a number of factors such as arterial transit time, inversion efficiency and the magnitude of the error [16]. These unsatisfied results may be caused by the inaccurate quantifications of CBF values [16]. Moreover, the CBF value could not represent the cellularity of gliomas.

There are some limitations in our study. First, the patient population was relatively small. Second, every grade gliomas contained different pathological subtypes, which would cause statistical deviations. In addition, the APT MRI used in this study was a 2-dimensional (2D) single-slice approach, which could not provide coverage of the whole tumor. In the future, we will enroll more patients with gliomas and investigate the differences between different pathological subtypes. Moreover, the APT MRI in monitoring the tumor recurrence and the treatment response should be investigated further.

In conclusion, APT MRI may be a superior imaging technique to DWI and 3D pCASL imaging in evaluating the grading and cellularity of gliomas. APT MRI is a promising method for predicting the grading and cellularity of gliomas.

## MATERIALS AND METHODS

### Patient population

A total number of 51 patients with pathologically proven gliomas were enrolled between August 2012 and November 2015 in this study. The inclusion criteria included: (a) MRI examinations performed on patients prior to treatments of gliomas; (b) the pathological diagnoses and Ki-67 indexes were acquired by surgical resections of gliomas and (c) APT MRI was performed at least 24 hours after the injection of contrast agents to minimize the influence of the gadopentetate dimeglumine in the APTw signal intensity [23]. The exclusion criteria included: (a) poor imaging quality due to motion artifacts; (b) solid tumor component unavailable for analysis ( < 20 mm2) and (c) WHO grade 1 gliomas because they were distinctive in the population (pediatric) and location (often posterior fossa) [24]. Finally, 5 patients with head movement artifacts and 2 patients with unavailable solid tumor components were excluded. A total number of 44 patients (21 males and 23 females; age range, 25-68 years; mean age ± standard deviation, 49 ± 11 years) with gliomas were included in the statistical analyses. Out of the 44 patients, 18 patients (41%) were confirmed with pathology to be WHO grade 2 gliomas, 10 patients (23%) were WHO grade 3 gliomas, and the remaining 16 patients (36%) were WHO grade 4 gliomas. The diagnoses of the patients included WHO grade 2 astrocytomas (n = 13), grade 2 oligodendrogliomas (n = 2), grade 2 oligoastrocytomas (n = 3), grade 3 anaplastic astrocytomas (n = 5), grade 3 anaplastic oligodendrogliomas (n = 2), grade 3 anaplastic oligoastrocytomas (n = 3), and grade 4 glioblastomas (n = 16).

This study was approved by the institutional review board of Zhengzhou University People's Hospital & Henan Provincial People's Hospital, and written informed consent was obtained from each subject before participation.

### MRI data acquisition

All patients were examined using conventional MRI, DWI and 3D pCASL imaging in a 3-T MRI unit (Discovery MR 750, General Electric Medical Systems, Milwaukee, Wisconsin, USA) with an eight-channel head coil (General Electric Medical System). Conventional fast spin-echo (FSE) sequences were performed including axial T1w image with a repetition time (TR) of 1593 ms, an echo time (TE) of 24 ms, a field of view (FOV) of 24 × 24 cm2, a matrix of 320 × 256 and a slice thickness of 4 mm, and axial T2w image (TR/TE, 4600 ms/110 ms; FOV, 24 × 24 cm2; matrix, 320 × 256; and slice thickness, 4 mm). The axial T1w sequence was repeated after intravenous administration of 0.01 mmol/kg gadopentetate dimeglumine (Magnevist, Bayer Schering Pharma, Berlin, Germany). DWI and 3D pCASL imaging were performed before the injection of contrast agents. DWI was obtained using a single-shot, echo-planar sequence in the axial plane (TR/TE, 4000 ms/112 ms; FOV, 24 × 24 cm2; matrix, 128 × 128; slice thickness, 4 mm; and slice number, 38). Two b values of 0 and 1000 s/mm2 (1 signal acquisition) were used in three orthogonal directions. The acquisition time for DWI was 48 seconds. 3D pCASL MRI was acquired using a FSE sequence in the axial plane (TR/TE, 4623 ms/10.5 ms; FOV, 24 × 24 cm2; slice thickness, 4 mm; slice number, 38; labeling duration, 2025 ms; and post-labeling delay time, 1525 ms). A total of 8 arms were acquired and each spiral arm included 512 sampling points in the k-space. The acquisition time for 3D pCASL MRI was 4 minutes and 24 seconds.

All patients were also examined using a prototype 2D single-slice radiofrequency-spoiled gradient echo APT MRI protocol in a 3-T MRI unit (Magnetom Trio, Siemens Healthcare, Erlangen, Germany) with a twelve-channel head coil (Siemens Healthcare). The APT MRI was obtained with a TR of 3200 ms, a TE of 2.87 ms, a FOV of 24 × 24 cm2, a matrix of 128 × 128, a slice thickness of 4 mm, 21 frequency offsets from -5 to +5 ppm with even intervals of 0.5 ppm. The unsaturated water image was acquired as first image in the APT MRI series. The presaturation was achieved using a train of five Gaussian-shaped radiofrequency saturation pulses with a continuous-wave amplitude equivalent of 2.0 μT. The total duration of saturation time was 995 ms followed by the readout of a single gradient echo image with centric reordering. The length of the each saturation radiofrequency pulse was 99 ms, and the gap between the pulses was 100 ms. The acquisition time was 1 minute and 45 seconds for a single slice. For each patient, two different slices of APT MRI were positioned at the same slice positions as the T2w images selected by the two radiologists.

### MRI data processing

Both DWI and 3D pCASL data were obtained and transferred to a workstation (Advantage Workstation 4.5, General Electric Medical System, Milwaukee, Wisconsin, USA) for processing.

The ADC value was calculated from the b values of 0 and 1000 s/mm2 by a mono-exponential model as follow [20]:

S(b)/S(0) = exp(-b × ADC),

where S(b) represents the signal intensity in the presence of diffusion sensitization, and S(0) represents the signal intensity in the absence of diffusion sensitization.

The CBF value was calculated by the following equation [25]:

$$\text{CBF}=\frac{\lambda \left(1-e^{\frac{-t_{sat}}{T_{1g}}}\right)}{\text{2}\alpha T_{1b}\left(1-e^{\frac{-\tau }{T_{1b}}}\right)}\frac{PW}{PD}e^{\frac{-PLD}{-T_{1b}}}$$

where t_sat_ represents the time of saturation performed before imaging (2000 ms), T1g represents the T1 of the gray matter (1200 ms), and T1b represents the T1 of the blood (1600 ms). α is the labeling efficiency (0.8), λ is the cortex-blood partition coefficient (0.9), and τ is the labeling duration. PW means the perfusion-weighted map, and PD means the proton density map. PLD represents the post-labeling delay time.

The APT data processing procedures were performed by Interactive Data Language (IDL, Research Systems, Inc., Boulder, Colorado, USA). The APT images were calculated by the magnetization transfer asymmetry between signal intensities of ± 3.5 ppm with respect to the water frequency using B0 corrected z-spectrum on a pixel-by-pixel basis [5]. The APTw value was calculated by the following equation [3]:

MTRasym(3.5 ppm) = MTR(+3.5 ppm) - MTR(-3.5 ppm) = Ssat(-3.5 ppm)/S0 - Ssat(+3.5 ppm)/S0

Where MTRasym represents the magnetization transfer ratio asymmetry. MTR represents the magnetization transfer ratio. Ssat and S0 represent the signal intensities measured with and without radiofrequency saturation, respectively.

The raw image data were organized into the z-spectrum, and then the z-spectrum was fitted using a 12th-order polynomial on a voxel-by-voxel basis. The unsaturated water image was used as a reference to calculate the APT effect. The B0 field inhomogeneity was calculated according to the deviation of the minimum of the fitted curve from 0 ppm. The correction of B0 field inhomogeneity in the APT MRI data-processing was used to make the results more reliably.

### MRI data analysis

The two radiologists, who were blinded to the pathological results of the tumors, independently analyzed all the APT, ADC and CBF maps by using ImageJ software (version 1.49b, NIH, USA). Before drawing the regions of interest (ROIs), the matrixes of T2w and CBF images were changed to 128 × 128. The two radiologists independently drew ROIs on the selected axial T2w images for each tumor. The ROIs were manually drawn around the entire solid part of the tumors. Areas of necrosis, cystic component, edema, cerebrospinal fluid or hemorrhage were excluded to ensure accurate measurements. Then, the ROIs on T2w images were copied to the APT, ADC and CBF maps of the same patient for the measurements.

### Histological processing immunohistochemistry

Specimens acquired from surgical resections were embedded in paraffin. Ki-67 immunohistostaining was conducted for quantification analysis. Slides were rinsed in phosphate buffer saline and blocked with 5% normal goat serum, followed by incubation with primary mouse monoclonal mouse anti-Ki-67 (ZM0165, Zhongshan Biotechnology Co., Ltd., Beijing, China) for 2 hours at 37oC. Slides were then incubated with horseradish peroxidase-conjugated secondary antibody diaminobenzidine (Fuzhou Maixin Biotechnology Development Co., Ltd., Fuzhou, Fujian, China) for 10 minutes at 37oC, and visualized with diaminobenzidine substrate (Fuzhou Maixin Biotechnology Development Co., Ltd., Fuzhou, Fuzhou, China).

### Immunohistochemistry data analysis

The two pathologists independently analyzed the Ki-67 index by using a HMIAS-2000 Medical Color Image Analysis System (Champion Image Engineering Co., Ltd., Wuhan, Hubei, China). They independently placed three different ROIs in the solid components of the tumor on the Ki-67 immunohistostaining map for each patient. The ROIs excluded the areas of necrosis, cystic degeneration or hemorrhage. The integrated optical density was measured for the Ki-67 index.

### Statistical analysis

All statistical analyses were performed with SPSS software (version 17.0, SPSS, Chicago, Ill). The mean results of APTw signal intensity, ADC value and CBF value for each patient measured by the two radiologists and the mean result of Ki-67 index for each subject measured by the two pathologists were used for statistical analyses. A Mann-Whitney U test was used for the comparison of each MRI parameter and Ki-67 index between the WHO grade 2, 3 and 4 gliomas. Bonferroni corrections were used for multiple comparisons. Spearman rank correlation analyses with 95% confidence intervals were used for assessing correlation coefficients between each MRI parameter and Ki-67 index in the gliomas. A Fisher's z transformation analysis was used for comparing the correlation coefficients. A ROC curve analysis with 95% confidence interval was used to assess the diagnostic utilities of APTw signal intensity, ADC value and CBF value in the grading of gliomas. The interobserver agreement for each quantitative measurement was assessed by using the intraclass correlation coefficient.
